# Hybrid lightweight vision transformers with attention mechanism for feature extraction and classification of product designs

**DOI:** 10.1371/journal.pone.0343510

**Published:** 2026-03-03

**Authors:** Abdul Wahid, Hikmat Ullah Khan, Anam Naz, Fawaz Khaled Alarfaj

**Affiliations:** 1 Department of Information Technology, University of Sargodha, Punjab, Pakistan; 2 Department of Computer Science, University of Wah, Rawalpindi, Pakistan; 3 Department of Management Information Systems, School of Business, King Faisal University, Al Ahsa, Saudi Arabia; Chunghwa Telecom Co. Ltd., TAIWAN

## Abstract

In modern consumer markets, product packaging strongly influences customer attention and buying decisions. Attractive and informative designs help brands stand out in competitive environments. Recently, Artificial Intelligence (AI) has been widely used to support packaging evaluation, especially for design analysis, personalized user experiences, and product recommendation systems. However, traditional deep learning models, such as CNN-based ResNet-50 architectures, often fail to capture long-range relationships and global visual context. These limitations reduce their effectiveness in complex visual tasks like packaging classification. To address this issue, this study investigates the use of vision transformer-based models for packaging design analysis. We propose LeViT, an efficient hybrid architecture that combines convolutional neural networks with vision transformers. This design enables the model to learn both local visual details and global contextual features. The proposed approach improves feature representation while maintaining computational efficiency. Experiments were conducted on an image dataset of packaging designs. The performance of LeViT was compared with state-of-the-art models, including CNN-ResNet-50, RegNet, and ConvNeXt. The results show that the proposed model achieves the highest classification accuracy of 95%, outperforming all comparison methods. These findings demonstrate the effectiveness of transformer-based architectures for packaging classification. The proposed approach offers practical benefits for retail analytics, brand assessment, and marketing decision-making.

## 1. Introduction

The study of brand attention and consumer perception plays a crucial role in marketing and product design, particularly in today's competitive retail landscape. With consumers exposed to an intensive variety of products both in physical stores and digital marketplaces, packaging design serves as a critical factor in attracting attention and influencing purchase decisions [1]. Traditional marketing theories emphasize the importance of visual appeal, including color schemes, typography, imagery, and branding elements, in shaping consumer perception [2]. However, the rapid advancements in Artificial Intelligence (AI) and deep learning have revolutionized the way researchers analyze and quantify consumer attention toward packaging [3]. Among these advancements, Vision Transformers (ViTs) have emerged as powerful tools for visual data analysis, offering a more sophisticated understanding of how design elements impact consumer engagement [4,5].

The application of deep learning in consumer-centric packaging design analysis offers several benefits. By leveraging AI-driven models, businesses can optimize product packaging to maximize consumer engagement and brand recognition. This technology facilitates predictive analytics, allowing brands to forecast market trends and consumer preferences based on visual attributes [6]. Moreover, AI-based models help identify the effectiveness of design elements, ensuring that packaging aligns with consumer expectations and enhances the overall shopping experience [7]. By incorporating deep learning techniques, organizations can bridge the gap between consumer psychology and design aesthetics, leading to data-driven decisions that enhance brand visibility [8]. Despite the advancements in AI-driven packaging analysis, several challenges remain. Traditional computer vision techniques such as Convolutional Neural Networks (CNN – ResNet-50 s) have been widely used for image classification and object recognition, but they often struggle with long-range dependencies and contextual relationships in complex visual data [9,10]. A significant challenge is the availability of high-quality visuals patterns [11], large datasets specifically tailored to packaging design evaluation [12]. Without a robust dataset, models may fail to generalize effectively, limiting their practical applications in real-world scenarios like healthcare, [13], marketing, and education.

In this research study, we address these challenges by integrating ViTs for analyzing consumer attention toward packaging designs. Unlike traditional CNN – ResNet-50 s, ViTs can capture global dependencies within images, making them more effective in identifying intricate visual patterns. Using available dataset which helps to enabling a comprehensive analysis of design elements that influence consumer perception. By training and evaluating ViT models on this dataset, we provide valuable insights into the key factors that differentiate successful packaging from ineffective designs. The following are the main research contributions in this study:

- Combines lightweight convolutional layers for local pattern extraction (edges, textures, fine details) with Transformer blocks for capturing long-range dependencies and global layout relationships in packaging designs.
- Proposal of hybrid LeViT for the identification of Top Product Packaging designs which considers attention bias, positional information and efficient classification by combining convolution-based and Vision Transformers
- Carrying out a comprehensive Experimentation which reveals that proposed LeViT, an advanced vision transformer model, achieves 95% accuracy, significantly outperforming traditional deep learning models (CNN – ResNet-50, RegNet, and Convex Net) in consumer-centric packaging classification.

In this study, section II defines the analysis of existing studies based on various deep learning methods from traditional to advanced techniques. Section III provides comprehensive research proposed methodology sharing phases to conduct this study. Section IV presents the insights into model predictive power by discussing results of model along their comparison with other studies. Lastly, section V shares the conclusion of this study along with future work.

## 2. Related work

Deep learning continues to drive advancements in consumer attention modeling and packaging classification, enabling improved brand recognition, automated packaging evaluation, and customer engagement. The Table 1 focuses on different models, methodologies, and datasets. There are several studies that use CNN architectures to enhance packaging image recognition. In this work, Gothai *et al.* [14], CNN method outperformed classification accuracy with the requirement based on manual preprocessing of images. Later, researchers used attention-based architectures for brand packaging recognition based on state-of-the-art ViT model, developed an improved Full Convolutional Network by Zhang *et al.* [15] for packaging design image segmentation to improve accuracy compared to the current model. Although the model was very computationally intense, resulting in it being nonviable for use in real time application.

**Table 1:** Summary analysis of existing studies.

Ref	Utilized Models	Dataset	Classes	Research Area	Limitations	Results (Acc %)
[18]	DNN	Food Packaging	9	Retail Expiry Date Recognition	Struggles with low-quality prints	91.8
[14]	CNN	Retail Image	10	Automated Retail Packaging Analysis	Manual image preprocessing required	89.3
[44]	DINO	Items Packaging	5	Item detection	High computational cost	89
[24]	EPformer (Efficient Transformer)	Retail Fisheye Image	10	Retail Product Detection	Difficulty in handling occlusions	92.3
[15]	Full Convolutional Network (FCN)	Packaging Image	8	Transformer-Based Packaging Classification	High computational cost	94.1
[16]	CNN + DNN, EfficientNet	Consumer Packaging	6	Emotion-Based Brand Packaging Recognition	Subjective label dependency	90.2
[22]	ViT	Infrared Defect Images	7	Defect Detection in Packaging	Requires high-resolution infrared imaging	93.4
[25]	ViT	Industrial Manufacturing	8	Industrial Packaging Quality Control	Requires specialized datasets	89.5
[27]	CNN Transformer Model	Brand Packaging	9	Logo Detection & Saliency Map Analysis	Requires labeled branding data	92.8
[19]	CNN, MobileNet v3	Artistic Packaging	4	Art and Texture Classification in Packaging	Limited scalability across styles	88.7
[20]	CAD- CNN	Graphic Design	10	AI-Driven Creative Packaging Design	Requires manual feature selection	93.5
[21]	CAD + 3D CNN Hybrid Model	Smart Retail	12	AI-Proposed Brand Packaging Design	High computational power required	92.1
[26]	DeiT	3D Printed Packaging	12	AI-Based 3D Printing Quality Control	Overfitting in small datasets	86.2

Emotion recognition is included in some studies within packaging analysis. Image emotion perception computing was applied by Yang *et al.* [16] based on deep learning to predict consumer reaction. However, the accuracy was highly dependent on subjective emotion labels, and so in generalization to any demographic was not possible. Recently, Yu *et al.* [17], integrated a hybrid CNN-transformer model for evaluating corporate brand packaging, with better classification metrics but relying on large amounts of labeled data to have reliable results. The main issue is food packaging recognition because the label variations are complex. As shown by Gong *et al.* [18], this framework builds a deep neural network-based system that can recognize expiry dates on the food packaging with high precision in structured labelling but poorly recognizes low quality printed texts. Like for packaging art painting recognition, Zeng *et al.* [19], also proposed a deep learning-based on texture for fine grained texture classification, but it is limited by its scalability in various packaging styles. It has also been used in the field of computer-aided package design with deep learning. In Chen *et al.* [20], one initiates a CAD based packaging design model that improves the design automation, but at the same time, it remains manual for the feature selection with respect to the best performance. Finally, Zhang *et al.* [21], built the brand packaging design evaluation based on AI, combining the CAD and deep learning model, which is better personalized branding with higher computational power demand. Industrial packaging design defect detection has been successful with transformers as demonstrated by Wei *et al.* [22], integrated a multi-scale reconstruction network for detecting infrared defects of packaging. The approach, however, needs high resolution infrared imaging, thereby preventing its practical real-world application. Zhang *et al.* [23] used CNN-Transformer Bidirectional Interaction Model for IC packaging material identification with a 91.6% accuracy.

However, the approach does not perform well in fine-grained texture differentiation in packaging materials. Introduced by Deng *et al.* [24], EPformer is a transformer-based model for detecting retail products in fisheye images allowing optimal distorted image classification but which struggles to deal with occlusions. Recent studies have examined the effectiveness of Vision Transformers in industrial packaging analysis. Furthermore, ViTs were found to be more effective in pattern recognition and classification at visual anomaly detection on quality control presented by Alber *et al.* [25] studies but need specific datasets to make the concepts practical. Singh *et al.* [26] integrated a real-time quality control system of 3D printed packaging using Data Efficient Image Transformers (DeiT), which fits in with small dataset scenarios. For branding and logo recognition for packaged product, the deep learning models have also been explored. Hosseini *et al.* [27] constructed a CNN – ResNet-50 -Transformer model for detecting and predicting logo and analyses the placement of logo in branding, which is convenient but costly in terms of labeling for massive branding datasets. The ViTs have been used in product packaging use cases, including agriculture and plant-based product packaging. However, supervised learning procedures fail to capture the characteristics and behaviors requisite for real-time automated discrimination of tea leaf packaging defects, while Lei et. al [28] have further used a Hybrid Fuzzy Based Transformer Model that have demonstrated a set of the defects. Finally, ViTs have been tested for the classification of retail product packaging by Nikolakis *et al.* [29] introducing vision Transformer for product packaging classification model with the resource of heavy computational power needed for scale deployment. ViT used by Prashanthi *et al.* [30] to investigate the fruit and vegetable disease classification but suffers with dataset noise and segmentation errors.

Recent advancements of smart-diagnostic and neural analysis models have shown the prospects of optimization algorithms and transformer structure in solution of biomedical signals. As an example, one of the proposed algorithms was a Modified Gray Wolf Optimization (MGWO-eP), which could be used to detect Parkinson’s Disease at the first stage, combining the ability to explore handwriting and speech data with the ability to select features, achieving 98.31% accuracy and outperforming traditional swarm-based algorithms [31]. Likewise, frequency-domain analysis with Variational Mode Decomposition (VMD) has been shown to be effective in the emotion recognition of EEG signals, with frequency band power characteristics obtained through a sliding window method being able to provide the random forest model with classification accuracies above 90%, demonstrating the discriminative strength of EEG-based intrinsic mode functions [32]. Building on these developments, the more recent reviews highlight the transformer-based architectures, such as Time Series Transformer, Vision Transformer, and hybrid versions, as having high potential in the EEG-based task, such as motor imagery and emotion recognition, as well as seizure detection, because of their increased ability to capture long-range temporal interactions [33]. When these studies are combined, they all point to the increasing tendency of neurocognitive and affective computing research to adopt hybrid and transformer-based frameworks. A multimodal network was suggested with dual head and spiral handwriting imagery and sequence data that were combined with an AttentionFusion module to detect Parkinson Disease in which a 92.36% accuracy was attained with an AUC of 0.943. The latest advances of deep learning have created crucial progress through the creation of multimodal and spectral images analysis [34]. A new SimPoolFormer architecture proposed a two-stream attention-in-attention Vision Transformer system that trains SimPool and ResMLP Neuromorphic units to provide highly efficient hyperspectral images classification and better accuracy than traditional CNN and ViTs [35]. Equally, Tri- CNN – ResNet-50 model has utilized a three-branch multi-scale 3D- CNN architecture that collectively learns spectral and spatial features with impressive classification on a variety of hyperspectral data [36]. Outside the area of image classification, new studies in Video Anomaly Detection (VAD) have been performed on multimodal fusion and weak supervision, such as a unified framework (UWS4VAD) that combines visual, audio, and textual modalities via pre-trained models such as CLIP and ViTamin encoders [37] to enhance anomaly interpretation and face the issue of class imbalance through dynamic sampling and curriculum learning policies [38]. Collectively these studies point to the trend of going to hybrid and multimodal structures that trade-off efficiency, interpretability, and cross-domain adaptability in visual data comprehension.

Furthermore, the recent research has proven the increasing role of deep learning and transformer-based architecture in medical and agricultural image analysis. As an example, CNN-based models have already demonstrated veritable success in identifying plant diseases, with MobileNetV2 and InceptionV3 scoring over 94% in maize disease classification, which proves the effectiveness of lightweight models in the field [39]. On the same note, CAD systems based on deep learning have shown great efficiency in healthcare diagnostic tasks, with InceptionV4 showing a 98.80% accuracy in lung cancer detection and a solid generalization performance across CT scans [40]. DenseNet169 demonstrated the best accuracy and computing efficiency in breast cancer detection based on mammograms, indicating its effectiveness in clinical applications in real-time [41]. In addition to the traditional CNNs, attention-enhanced ConvNeXt networks that combine block and grid attention networks have been demonstrated to have outstanding performance in crack detection, with 99.98% accuracy, and their interpretability can be enhanced to support structural health monitoring [42]. As the direction of this trend, new models based on transformers, including Swin-Small and Swin-Large, have become new paradigms in classifying brain tumors, and Swin-Small with an accuracy of 89% above consider computational efficiency, which is paramount to the accuracy-scalability balance in contemporary diagnostics devices [43].

Overall, the analyzed literature highlights the accelerated development of deep learning models – beyond CNNs to transformer-based hybrids – in promoting visual recognition tasks in different applications. These models always show that fusion, attention mechanisms, and arch efficiency are the key to the high diagnostic accuracy. Based on these, this paper uses the hybrid LeViT architecture to trade-off between computational efficiency and global contextual knowledge to overcome major shortcomings of the conventional CNN-based systems.

The current developments of intelligent fault diagnosis and predictive maintenance have more based on improving model adaptability, interpretability and transferability in complex industrial environments. The study based on ensemble domain adaptation network [45] suggested the combination of ensemble learning and weighted balance domain adaptation to produce strong and high-accuracy pseudo labels to support unsupervised domain adaptation and outperforms and surpasses the previous ideas in cross-condition and cross-device bearing fault diagnosis. Similarly, another study [46] integrated a multi-source adversarial online knowledge distillation framework of remaining useful life (RUL) prediction across heterogeneous machines, which can dynamically apply knowledge transfer to multi-level domain adaptation and overcome extreme drifting distribution issues and scarcity of labeled samples. Moreover, adaptive frequency attention-based interpretable transformer network [47] utilized to integrated multiscale convolutional embeddings with frequency-aware attention to enhance both diagnostic accuracy and interpretability paired with few-shot learning. Taken together, these studies indicate a definite shift to models that can be more performance-adaptive-transparent, but many of them are limited by the high costs of computation and reliance single dataset – which limits the model interpretation and generalizability.

## 3. Proposed research methodology

**Fig 1:**
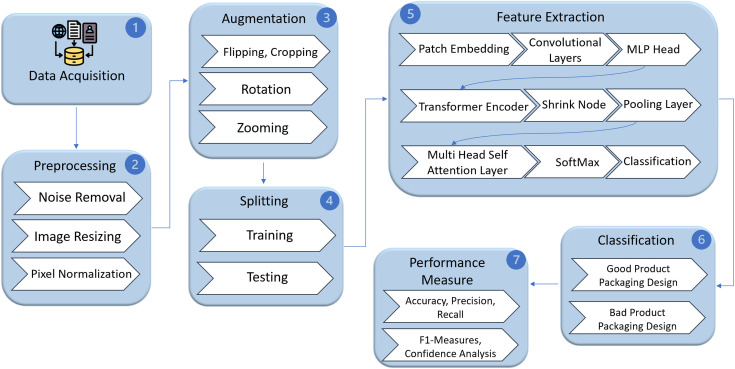
Sequential steps of the proposed methodology.

The research has a systematic approach to the correct recognition and classification of product packaging designs through ViT-based models. The general workflow of the project involves data collection, data preprocessing, model selection, model training, model evaluation and performance validation as shown in Fig 1. All the steps will be aimed at providing a stable learning process and equitable comparison of performances. The main novelty of the framework consists in the fact that LeViT, the hybrid CNN – ResNet-50 -ViT architecture, which can effectively merge the extraction property of CNN – ResNet-50 with the global attention property of transformers, is proposed. This combination makes effective representation learning since it can capture both fine-grained spatial features as well as long-range relationships in images, and it improves the accuracy of classification. Moreover, several deep learning baselines, such as traditional CNN – ResNet-50 and transformer forms, were used to conduct comparative studies, and the superiority of the proposed LeViT model was proven. This is the advantage of the framework, it trades off computational efficiency and model accuracy in the most effective way possible, showing a balanced design with the ability to handle complex visual recognition tasks and remain scaled and interpretable.

### 3.1. Phase I: comprehensive data analysis

The first phase of this study involves a thorough analysis of the dataset, which is crucial for the characterization of the dataset as well as verifying its suitability for training deep learning models. This dataset has 2 classes represent as ${\text{N}}_{\text{good}}=\;{\text{N}}_{\text{bad}}=\;\frac{\text{N}}{\text{2}},$ where N is the total number of images in the dataset, with equal number of examples belonging to every class to maintain balance. Dataset collected from various sources such as consumer surveys, e-commerce platforms, designer portfolios for a wideness in terms of colors, textures and structural patterns of packaging designs. The class ratio is calculated to confirm balance, ensuring that dataset does not suffer from imbalance issues. In this study, the terms good and bad packaging refer to the overall presentation quality of product packaging, where good indicates products placed in well-designed, visually balanced, and professionally structured packages, whereas bad denotes roughly arranged, poorly aligned, or visually inconsistent packaging styles.

Methods: This study did not involve the creation or collection of new data. Instead, it utilized an open-source dataset that is publicly available on GitHub for academic and research purposes. All data used in this research comply with the terms and conditions of the original data source. Ethical approval was not required, as the study relies solely on publicly available, non-human subject data. All analyses were conducted strictly within the permitted scope of academic research usage, ensuring transparency, reproducibility, and compliance with the original data source policies.

### 3.2. Phase II: data preprocessing

Preprocessing stage emphasizes the remuneration of noise, augmentation and normalization of data. Noise removal is used to remove distortions and artifacts in raw images [48]. Images are smooth with the help of Gaussian filtering, and salt-and-pepper noise is removed with the help of median filtering, which replaces pixel values with the median of their neighbors. Such filtering methods can be used to maintain significant edges and minimize undesirable variations [49].

To increase strength even more, geometry data augmentation is used. This involves horizontal and vertical flipping that enhances the variation of data and contributes to the reduction of overfitting. Horizontal flipping will enable the model to acquire orientation-invariant features, which will enhance its capacity to generalize new packaging designs. Moreover, images are resized to 224 224 with bilinear interpolation to ensure that the image size is the same in the entire dataset. The photometric augmentation, too, is used to reproduce changes in the lighting conditions, which makes the model better adapted to the real-world conditions [50]. These transformations ensure that the dataset is robust to real-world variations and improves the model's generalization capabilities.

### 3.3. Phase III: feature extraction and classification

During this phase, deep learning models are used to classify between good and bad feature packaging designs using feature extraction and classification. The LeViT proposed model combines the advantages of CNN-ResNet-50 and ViTs to strengthen within local and global features.

#### 3.3.1. LeViT proposed model.

**Fig 2:**
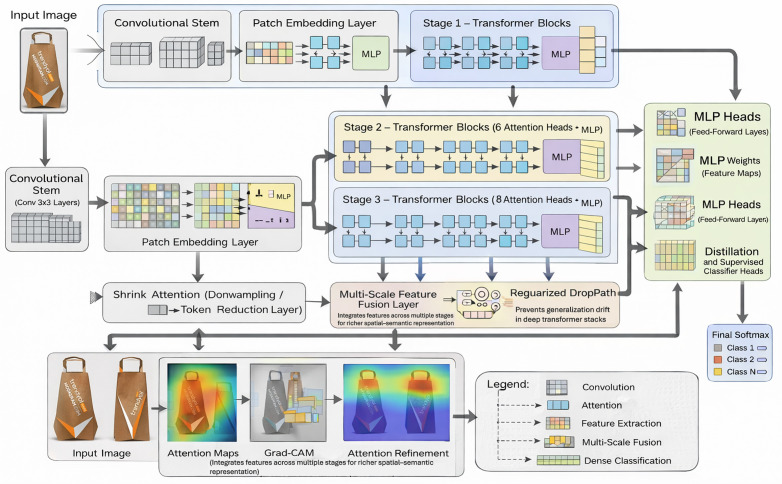
Architecture analysis of LeViT model.

To be classified, a hybrid deep learning model is proposed LeViT, this model is a mixture of both Convolutional Neural Networks (CNNs) and Vision Transformers and is used to classify the product packaging design as either good or bad. The general structure of the suggested model is presented in Fig 2. LeViT is created to extract both local and global features, which are sides and textures and layout and design patterns respectively. This renders it to be effective in performing intricate and fine grained visual data examination tasks [51].

It starts with the architecture using CNN backbone, which extracts hierarchical features of the input images. The model identifies the relevant local patterns in the packaging designs by using several convolutional blocks, with ReLU activation functions. These feature maps highlight edges, textures and structural details that are very important in packaging evaluation. Passing the extracted feature maps through transformer layers results in global context modeling [52]. The feature maps are then split into patches that do not overlap. The patches are flattened and mapped into a lower-dimensional space with a learnt projection matrix. It transforms the two-dimensional feature maps into the sequence of the embeddings, which can be transformed into the transformer processing. The transformer encoder comprises of series of layers of multi-head self-attention (MSA) networks and feed-forward networks (FFNs). The mechanism of self-attention allows the model to obtain long-range dependencies by obtaining the relationships between all the patches of images [53]. Lastly, a classification head transforms the feature representation obtained with the help of a fully connected layer and a SoftMax activation function. This gives a probability distribution of both target classes. The final prediction is the one that has been chosen by the highest probability. Table 2 summarizes the entire flow of the improved LeViT architecture. In general, LeViT is a successful combination of CNN based local feature extraction and transformer based global reasoning, which can be successfully used in consumer centered analysis of packaging design.

**Table 2:** Architectural components and functional description of proposed model.

No.	Layers	Functionality
1	Convolutional Stem (Conv 3 × 3 Layers)	Extracts low-level spatial features and local textures while reducing input resolution efficiently.
2	Patch Embedding Layer	Converts convolutional feature maps into tokenized patch embeddings suitable for transformer processing.
3	Stage 1 – Transformer Blocks (4 Attention Heads + MLP)	Captures early contextual relationships with lightweight multi-head self-attention and feed-forward layers.
4	Stage 2 – Transformer Blocks (6 Attention Heads + MLP)	Learns intermediate-level semantic representations with increased attention capacity.
5	Stage 3 – Transformer Blocks (8 Attention Heads + MLP)	Models high-level global dependencies and complex feature interactions.
6	Shrink Attention Layer (Downsampling / Token Reduction)	Reduces token dimensionality to improve computational efficiency while preserving salient information.
7	Multi-Scale Feature Fusion Layer	Integrates representations from multiple stages to enrich spatial–semantic feature diversity.
8	Attention Refinement Layer (ARL)	Recalibrates attention weights to emphasize discriminative regions and suppress irrelevant features.
9	Regularized DropPath Layer	Improves generalization by randomly dropping transformer paths during training to prevent overfitting.
10	MLP Heads (Feed-Forward Layers)	Performs nonlinear feature transformation and dimensional projection before classification.
11	Global Average Pooling Layer	Aggregates spatial features into a compact global representation.
12	Distillation and Supervised Classifier Heads	Supports both teacher-student knowledge distillation and standard supervised learning.
13	Final SoftMax Output Layer	Produces normalized class probabilities for final prediction.

#### 3.3.2. Baseline models.

Whether the proposed Vision Transformer-based model is effective or not, the model is compared with some of the most popular deep learning constructions that are typically utilized in the task of image classification. They are CNN- Resnet-50 used to extract local features, RegNet used to scale the structured network, and ConvNeXt, which involves a combination of convolutional layers and modern principles of architectural design. These baseline models are used as reference points on how to assess the progress of the classification accuracy, features representation and efficiency in computations. CNN-ResNet-50 has extensive applications in image-related activities like consumer attention and packaging analysis. It performs well to extract spatial hierarchies and detect visual properties like logos, colors, text positioning etc. [54]. Nevertheless, CNN- ResNet-50 does not perform as efficiently as others, and it has a weakness since it cannot capture long-range dependencies and global contextual relationships, which prevents its application in complex packaging designs [55].

Regularized Neural Network (RegNet) is a structured deep learning model for efficient scaling and optimized architecture learning. Unlike manually designed layers in traditional CNN, RegNet takes the form of a regularized pattern in feature channels and depth of blocks, to automatically discover the best architecture for optimal performance. Principled scaling is used to build the model with the feature learning balanced across layers [56]. RegNet achieves superior accuracy to complexity ratio with dynamic adjustment of width, depth and group width. It has a special property to serve ideally image classification, object detection and consumer attention analysis about packaging design, where accuracy and efficiency can be balanced, while keeping it scalable in real-world applications. A hybrid deep learning model consisting of an adaptation of convolutional CNN – ResNet-50 and the global contextual understanding of ViT is called Convex Net Model. Convex differs from traditional CNN in that it dynamically captures long range dependencies, among various other things, by employing convolutional layers for early feature extraction and multi head self-attention (for global feature representation) [57]. The model consists of dual branch architecture where one branch is learning spatial hierarchies using convolution and the other one uses self-attention to model contextual dependence.

Consequently, Convex can retain detailed fine-grained information and yet still capture global dependent, making it very powerful in image classification and placement design analysis and consumer attention modeling [58]. Although real-time versions of Convex are suitable for complex visual tasks, they are computationally expensive, and thus optimized hardware is required.

### 3.4. Machine requirements

The training and deployment of Vision Transformer-based models and to do it efficiently, a high-performance computing environment is needed. A system configuration, as shown in Table 3, consists of a multi-core processor, a high-end graphics card, adequate memory and advanced cooling system to accommodate lengthy training processes. Model implementation is performed using deep learning systems like PyTorch or TensorFlow and the experiments can be run on any standard operating system. Large-scale experimentation is also supported by high-speed internet connectivity and resources based on clouds [59].

**Table 3:** Machie requirements for implementation.

Component	Specification	Justification
Processor (CPU)	Intel Core i9 (12 + Cores, 3.5GHz+)	High-speed computation for model training tasks.
Graphics Card (GPU)	NVIDIA RTX 3090 / Tesla V100	Essential for model training and inference acceleration.
RAM (Memory)	32GB + DDR4/DDR5	Handles large-scale image data and deep learning model execution.
Storage	1TB NVMe SSD	Fast read/write speeds for model checkpoints.
Operating System	Windows 11	Compatibility with deep learning frameworks
Deep Learning Framework	PyTorch 2.0 / TensorFlow 2.10+	Supports models with optimized GPU acceleration.
Power Supply (PSU)	800W+ (Platinum Rated)	Ensures stable power for high-performance computing components.
Cooling System	Liquid Cooling or High-Performance Air Cooling	Prevents overheating during training sessions.
Networking	High-speed internet (1Gbps+)	Facilitates dataset downloading, cloud-based model training, and updates.

### 3.5. Performance evaluation measures

The standard metrics were implemented to extensively evaluate the performance of the proposed model to determine its classification performance aspects [60]. Among all metrics defined in Equation 1–4, Accuracy remains fundamental because it reveals how many instances a model labels correct while also determining how many exist in the sample data. Model precision describes the ratio of correct positive predictions by counting true positives against the combination of true positives and false positives. A substantial recall score demonstrates that the model successfully reduces instances of detection omission [61]. To balance precision and recall through the F1-score calculation which uses harmonic mean mathematics to produce a unified metric which evaluates false positives and false negatives. This metric proves valuable for cases that require equivalent prevention of false positives together with false negatives. To evaluate the model’s ability to accurately identify negative cases the ratio between true negatives becomes a vital metric called specificity in this way a model demonstrates superior performance in its ability to distinguish good and bad packaging designs with a high AUC metric [62]. These multiple evaluation metrics assess a model from diverse perspectives enabling robust performance in all classification aspects.


$$\begin{array}{c} \text{Accuracy}\;=\frac{\text{TP}+\text{TN}}{\text{TF}+\text{FN}+\text{FP}+\text{TP}} \end{array}$$ (1)

$$\begin{array}{c} \text{Precision}\;=\frac{\text{TP}}{\text{TP}+\text{FP}} \end{array}$$ (2)

$$\begin{array}{c} \text{Recall}\;=\frac{\text{TP}}{\text{TP}+\text{FN}} \end{array}$$ (3)

$$\begin{array}{c} \text{F1}-\text{Score}\;=\frac{\text{2}(\text{Precision}*\text{Recall})}{\text{Precision}+\text{Recall}} \end{array}$$ (4)

Where TP, TN, FP, FN stands for True Positive, True Negative, False Positive, and False Negative.

## 4. Experiments and observations

The proposed model performance is compared to the performance of baseline architectures in the form of several metrics, such as accuracy, precision, recall, F1-score, and computational efficiency. The suggested model never fails to be superior to traditional CNN-based models in classification accuracy and generalization. Other tests assess training convergence, behavior loss, inference speed and confidence scores. These findings prove that the developed Vision Transformer-based solution is strong and can be applied to practical product packaging recognition tasks.

### 4.1. Results of LeViT model

The LeViT model was trained and evaluated for consumer-centric packaging classification with high accuracy and computational efficiency. The resulting classification report display in Table 4 shows the performance of the model at distinguishing good packing design from bad packing with great predictive power. With an overall accuracy of 95%, the precision, recall, and F1 score is greater than 90% for each of the classes. Good packing can achieve 98% precision, which means no false positive in this category. However, its recall is only 90%, which is slightly lower than the expected 100%, due to the presence of some false negatives. On the other hand, it has a 97% recall, meaning all bad packaging instances are classified almost correctly, but a precision of 91%, implying some bad classified cases. Therefore, the high macro and weighted averages of 95% indicate that the model is balanced and performs well in both classes.

**Table 4:** Classification results using the proposed model.

Classes	Accuracy	Precision	Recall	F1-Score
Good Packaging Designs	95	98	90	95
Bad packaging Designs	95	91	97	95

**Fig 3:**
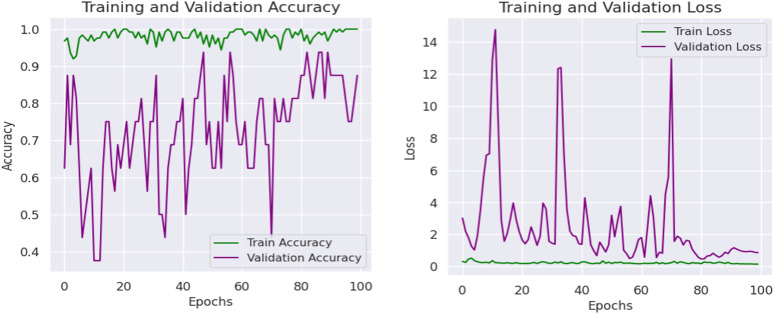
Loss and accuracy graphs of proposed model.

The validation accuracy graph shown in Fig 3 shows that the validation accuracy follows closely between 70 and 95%. Training accuracy is close to 100%. These findings suggest that while the model generalizes well it does so with a certain amount of variation in validation performance which may be caused by over-fit (including very few validation samples). The loss graph also shows that the training loss is very low, and validation loss jumps around at some points, which makes me think that either training loss or validation loss is overfitted or there is high variance in validation set. During training, validation loss does stabilize in the end, and it gradually learning converged.

**Fig 4:**
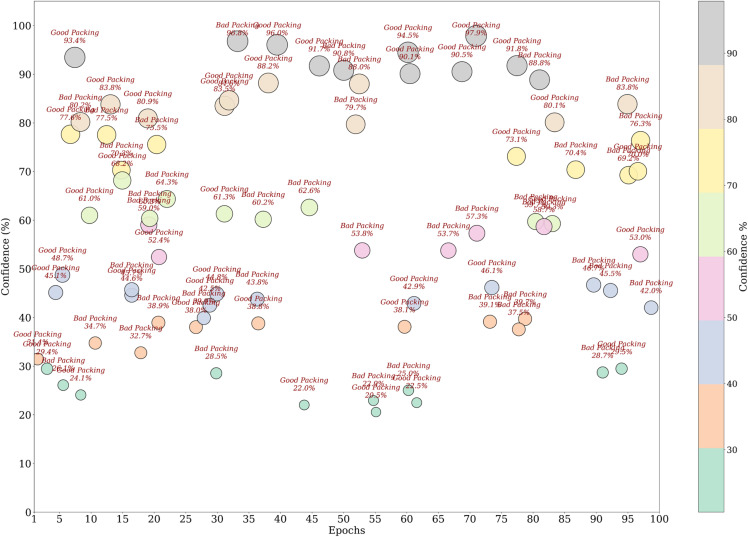
Confidence score analysis among samples.

**Fig 5:**
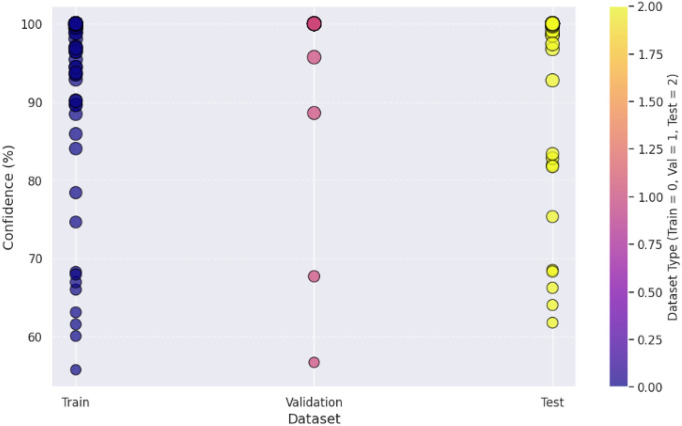
Confidence score analysis among dataset splitting.

The visualization of the confidence score in Fig 4 for the model shows that it tends to predict the packaging classes with varying levels of certainties. In fact, the most predictions have more than a 90% confidence, some samples predict in 60–80% confidence range, which means there are some ambiguous cases where the model does not have a strong enough confidence level to guarantee a correct prediction. This can show that there is improvement over time in the confidence scores for the scatterplot across epochs, which later epochs provide a higher prediction which clearly shows that model fine tuning leads to more stable predictive performance. The dataset split analysis in Fig 5 is a comparison of the confidence levels across training, validation and test sets. Strong internal feature learning is reflected in predictions in the training set that are very close to 100% confidence. However, most of the validation and test set confidence values are comparatively more varied, classes can be more confidently validated and tested. The LeViT model optimized to a stable convergence and high performance by ensuring that the hyperparameters were carefully optimized. Adam optimizer with the learning rate used with 32 batch size and 100 training epochs, as displayed in Table 5. The reason why dropout and weight decay were used was to avoid overfitting and ReLU activation and SoftMax output were used to make sure that features learn effectively and the separation of classes. Early termination and validation check were also used to ensure generalization and computational cost-effectiveness of the models.

**Table 5:** Hyperparameter settings of proposed model.

Parameter	Description	Values
Batch Size	Number of samples processed per iteration.	32
Patch Size	Size of image patches processed by the transformer.	16 x16
Embedding Dimension	Size of token embeddings.	192
Number of Layers	Total number of transformer blocks.	31 (including MLP heads)
Number of Heads	Number of attention heads per transformer block.	6
MLP Dimension	Size of fully connected layers in MLP.	3072
Activation Function	Type of activation used in the model.	GELU – Final SoftMax
Optimizer	Algorithm to adjust model weights.	AdamW
Epsilon	Small value to prevent division by zero.	1.00E-05
Dropout Rate	Probability of dropping a neuron during training.	0.1
Epochs	Number of complete training iterations.	100
Learning Rate	Step size for updating model weights.	0.00005
Beta 1 & Beta 2	Momentum parameter for optimizer updates.	0.9 & 0.999
Weight Decay	Regularization to prevent overfitting.	0.01
Learning Rate Scheduler	Gradual decay to prevent overfitting	Cosine Annealing
DropPath Rate	Prevents overfitting in deep transformer stacks	0.1
Label Smoothing	Reduces model overconfidence	0.1
Dropout in FC Layers	Dropout applied in fully connected layers.	0.1
Brightness Range	Randomly adjusts image brightness during augmentation.	0.2 to 0.4
Patience	Number of epochs without improvement before stopping.	3
LeViT Transfer Learning	Use of pre-trained models.	LeViT-192

**Fig 6:**
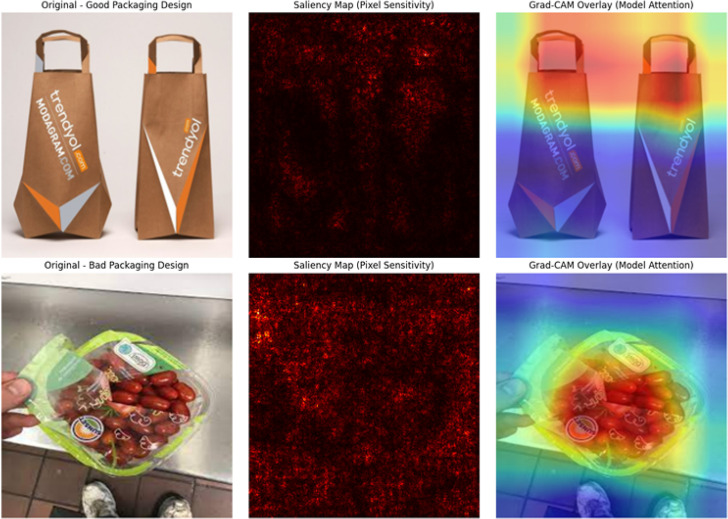
Saliency and Grad-CAM comparison between good and bad packaging designs using LeViT Model.

Fig 6 depicts the analysis of the interpretability of the proposed LeViT based model by visualizing the saliency and Grad-CAM with respect to good and bad packaging design. The saliency maps demonstrate pixel sensitivity, i.e., how the model reacts to parts of visual saliency, whereas the Grad-CAM overlays represent the global spatial attention patterns that the hybrid CNN-Vision Transformer system has learnt. Whereas good packaging is concerned, the LeViT model apparently narrowed down to the structured design aspects like the positioning of logos, color composition, and geometry orientation, which attests to its competence to embrace both the local and the global design coherence. On the bad packaging, on the other hand, the focus of the model was diffused all over uneven spaces, such as the reflection, the background sound, and the imprecision of the textual positioning, which indicated that it was aware of the visual inconsistency and a lack of harmony in design. These visualizations show that LeViT uses convolutional feature extraction with transformers based global attention to be able to interpret complex visual features concerning aesthetic quality and consumer perception. The high level of focus in good designs and diffuse attention in bad ones prove the interpretive ability of the model and proves its quality in design assessment.

**Fig 7:**
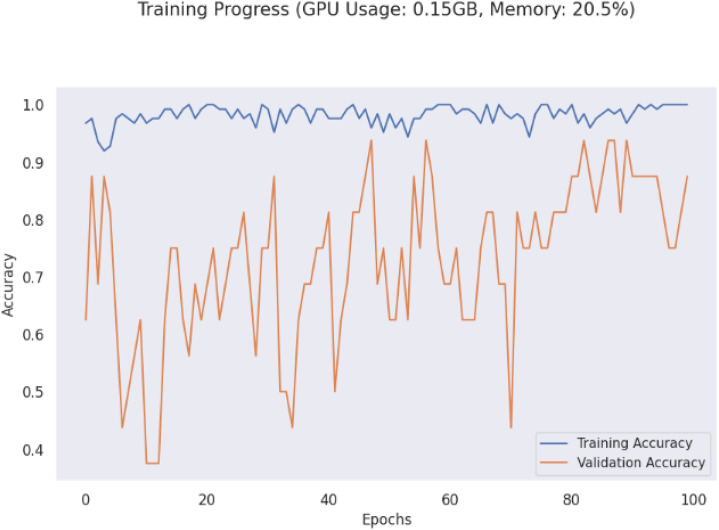
Computational resource of proposed model.

Furthermore, the LeViT model shows very good resource consumption in intra GPU memory and computational usage graph in Fig 7. It has low memory utilization of almost 0.15GB GPU usage with 20.5% memory, thus it is suitable for real-time deployment. LeViT is a transformer-based backbone, yet its computational footprint is manageable, which makes it lighter than ViTs. The predictive power of the model is strong, with accuracy of 95% on the LeViT model suggests that it is effective in the tasks of packaging classification. Strong generalization capabilities are shown by its high confidence scores and stable classification metrics, except for some validation loss fluctuations while some minor optimization could still be made. The model achieves considerable performance to well address fine grained visual details while computationally efficient.

### 4.2. Comparison of existing models

The comparison with a commonly used deep learning architecture in image classification, CNN – ResNet-50, and consumer attention model, RegNet, and Convex Net, is conducted to evaluate the effectiveness of the LeViT model. Local features can be extracted better than CNN – ResNet-50 s, but long-range dependency cannot. The scalability and efficiency come from the dynamic support for network depth and width adjustment that is inherent in CNN – ResNet-50 in RegNet, which overcomes CNN – ResNet-50 limitations. Using a fusion of CNN – ResNet-50 feature extraction along with Vision Transformer based global attention, Convex Net favors CNN – ResNet-50 based local information and combines with the Transformer based global dependency for maximum effectiveness. Using accuracy, F1-score, loss trends, confidence scores and computational efficiency, we evaluate the performance of LeViT against these models in packaging classification and in generalization performance. We discuss the findings based on the performance results of the model.

#### 4.2.1. Results of CNN – ResNet-50 model.

**Fig 8:**
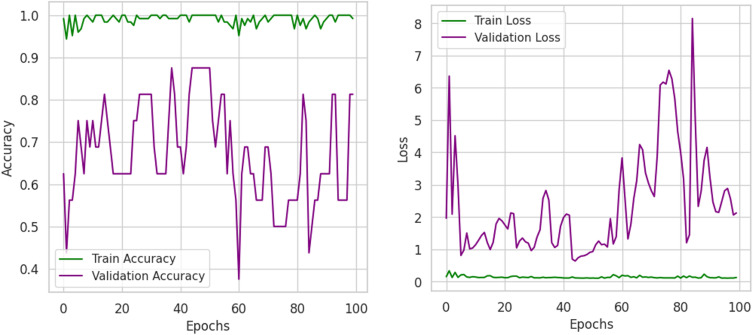
Loss/accuracy graphs of CNN – ResNet-50 model.

The CNN – ResNet-50 model shows the accuracy of 70% which is significantly less than the LeViT model. Good packaging model has higher precision 83% but suffers from recall 53% which means it misclassified thousands of Good packaging instances to Bad packaging. On the contrary, Bad Packing class has a high recall of 79%; it correctly marks worst packaging cases, but with low precision 63% as it has high false positive rate. The model's ability to classify good packaging is 59%, and bad packaging is 76%; thus, it gives an unbalanced performance indicate that the CNN – ResNet-50 model fails to yield consistent accuracy on the prediction of good packaging instances. The accuracy graph in Fig 8 shows that the training accuracy stays perfectly high (~100%) but validation accuracy very often exceeds 50% and at some points is noticeably lower than 100%. Thus, this implies overfitting, meaning that the CNN – ResNet-50 model has learned on the training specific features, but fails to generalize to unseen validation data. Similarly, the validation loss in the loss graph is very high and so the model appears to be unstable in the classification task. This pattern of irregular learning indicates the need for additional methods of regularization, increased depth of the architecture, or additional training data forced to the CNN – ResNet-50 to improve stability and generalization.

**Fig 9:**
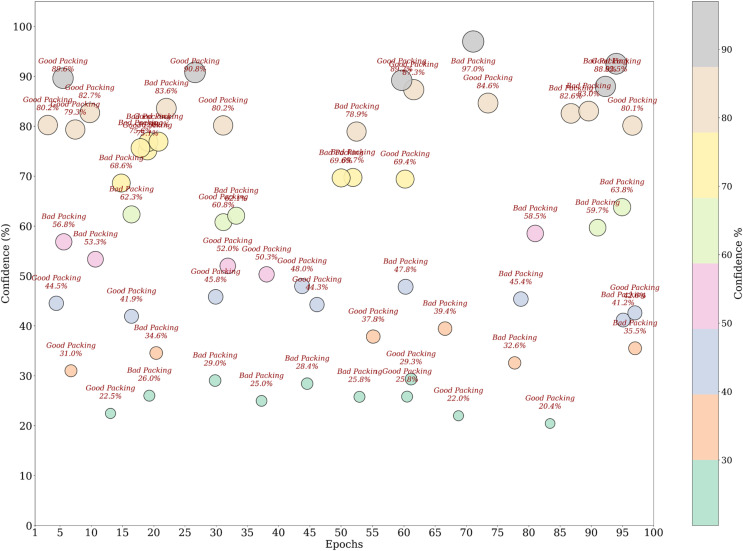
Confidence score analysis among samples.

**Fig 10:**
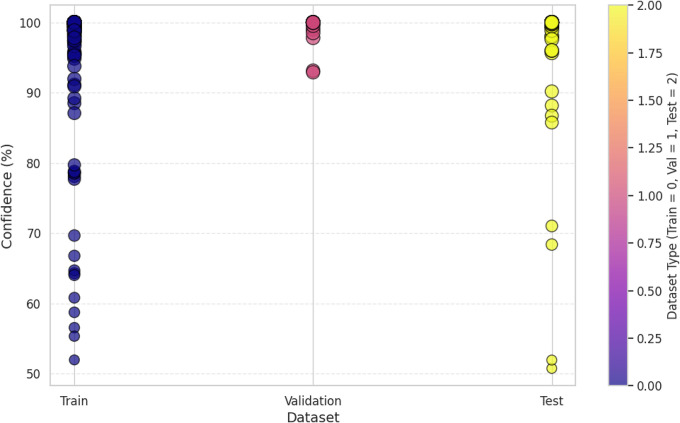
Confidence score analysis among dataset splitting.

The confidence level visualization in Fig 9 shows that CNN – ResNet-50 does predict some samples at high confidence (> 90%) while other samples are around 60–70% which might indicate uncertainty in taking a decision. Next, the dataset splitting analysis in Fig 10 confirms further that the training confidence is a lot more than validation and test confidence, and this in fact proves that the model is overfitting. However, such fluctuations in the confidence of the CNN – ResNet-50 may result in the misclassifying of the packaging designs in the real world, making the CNN – ResNet-50 less appropriate for the purpose of high stakes decision making in consumer centric packaging evaluation. Finally, CNN – ResNet-50 exhibits a lack of robustness since the fluctuating confidence scores and many false negatives, which suggest that CNN – ResNet-50 is not reliable to model real world consumer attention.

#### 4.2.2. Results of RegNet model.

A comprehensive analysis of the RegNet model’s classification report in Table 6 indicates a final accuracy of 85%, comparable to CNN – ResNet-50 but significantly lower to LeViT good packaging designs (and the model) achieved precision of 78% and recall of 79%, resulting in an F1 score of 87%, an indication of high precision and recall balance. In bad packaging designs, recall drops to 73%, which means accuracy of 86% but again the F1-score becomes 83%. Here, we see that sometimes bad packaging gets considered as good, although this model keeps a good overall classification performance.

**Table 6:** Classification results using RegNet model.

Classes	Accuracy	Precision	Recall	F1-Score
Good Packaging Designs	85	78	79	87
Bad packaging Designs	85	86	73	83

**Fig 11:**
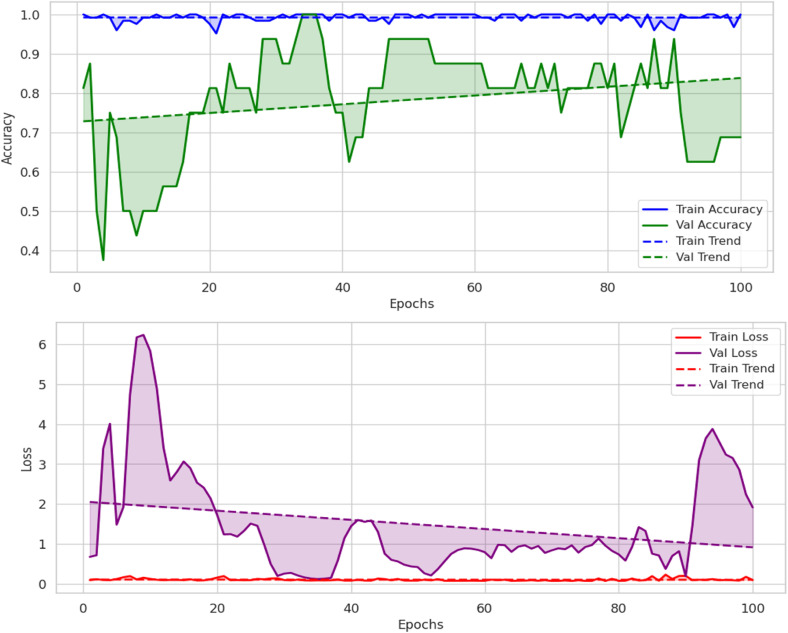
Loss and accuracy of model across training and validation sets.

The training and validation accuracy graph plots in Fig 11, ResNet’s near perfect training accuracy and its validation accuracy in a) around 70–85%. This pattern does hints at some overfitting behavior, as the model is doing very well on the training set, but the validation data is quite variable. This is confirmed more by the loss graph in b), which shows that the training loss is quite low and stable, but a validation loss can spike sometimes, meaning that sometimes it does not generalize enough. This demonstrates in these results that structural patterns in packaging can be learned by RegNet but that some regularization techniques may be needed to have better generalization.

**Fig 12:**
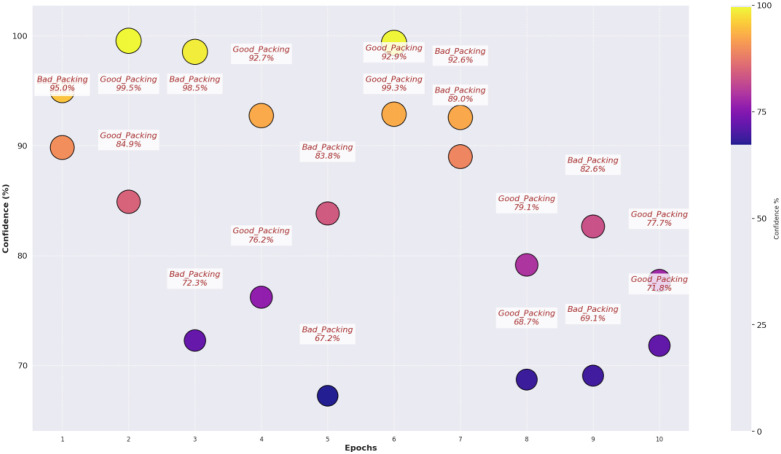
Confidence score analysis among samples.

**Fig 13:**
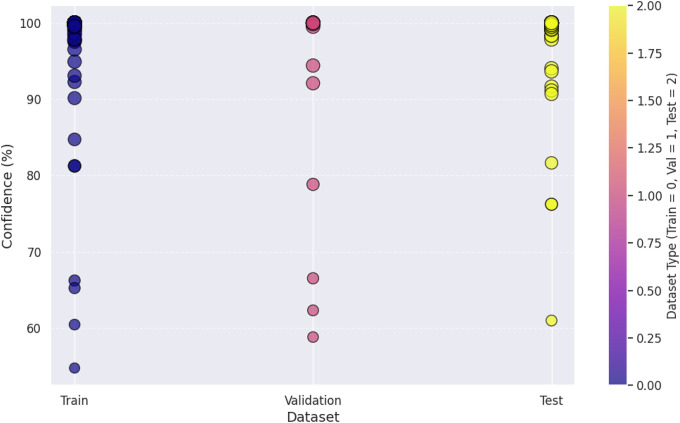
Confidence score analysis among dataset splitting.

The confidence score analysis in Fig 12 shows that RegNet makes predictions with great degrees of certainty for most cases; numbers indicating high 90’s confidence as defined. But some samples are in the 70–80%, especially in the bad packaging zones, indicating possible error in some cases. The confidence analysis of the dataset splitting shown in Fig 13 concludes that the training predictions are reliably confident, while the validation and test sets have a lot more varied levels of confidence, indicating the presence of overfitting and the need for further fine tuning.

On a more quantitative side, RegNet outperforms CNN – ResNet-50 substantially both in prediction accuracy (85%), recall, and in a better definition of a classification boundary. But it is still behind LeViT to a degree in handling bad packaging designs. Analysis of the confidence score indicates that RegNet is very confident about its predictions and has borderlines to deal with misclassification. Some overfitting is hinted at by the high training accuracy vs. fluctuating validation accuracy, but RegNet generally has a good tradeoff between efficiency, and scalability, and generalization, and it is therefore a solid candidate to package as a classification network.

#### 4.2.3. Results of Convex Net model.

In Table 7, the Convex Net model achieves an overall accuracy of 87% outperforming CNN – ResNet-50 and RegNet and comes close to LeViT Good Packaging Designs and Bad packaging Designs have both strong predictive ability with precision, recall, and F1-score. The combined precision and recall of Good Packaging Designs was 89%, the F1-score was 86%, and with regards to a relative balance of precision and recall, these were pleasing numbers, but they did lose some good packaging samples in the process. In Bad packaging Designs, the precision, recall and F1-score were 84%, 88% and 85% respectively, which was superior to Good Packaging in recall, making bad packaging designs detected. This finds that packaging analysis with Convex Net balances precision and recall nicely, and so is a robust classifier for this task.

**Table 7:** Classification results using Convex Net model.

Classes	Accuracy	Precision	Recall	F1-Score
Good Packaging Designs	87	89	83	86
Bad packaging Designs	87	84	88	85

**Fig 14:**
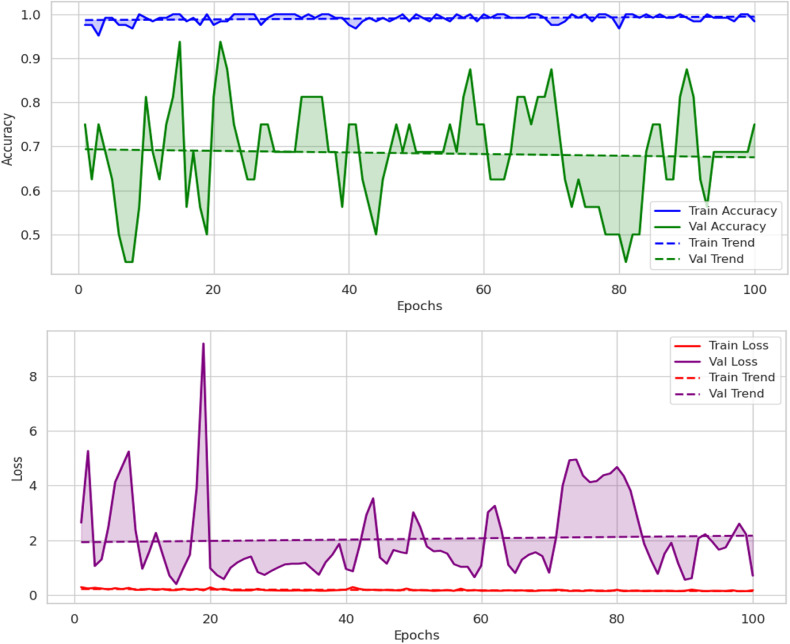
Loss/accuracy of model across training and validation sets.

**Fig 15:**
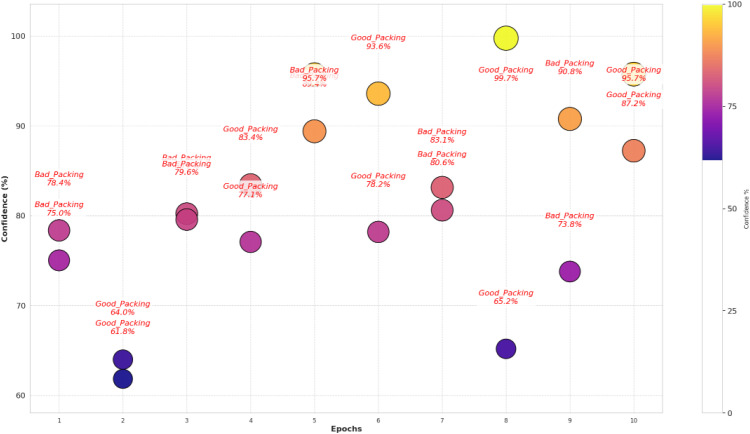
Confidence score analysis among samples.

**Fig 16:**
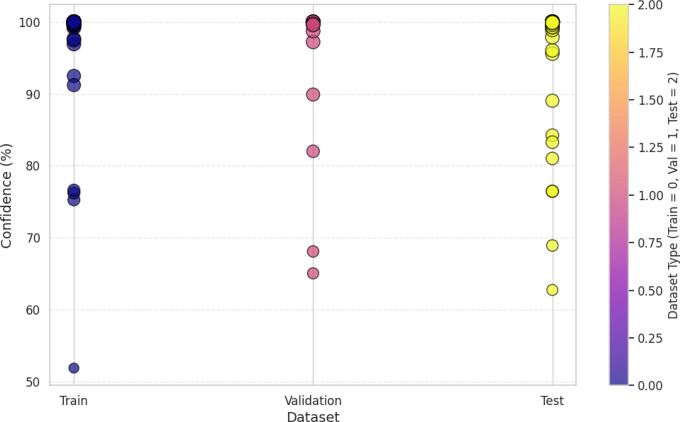
Confidence score analysis among dataset splitting.

The training and validation loss/accuracy graph in Fig 14 depicts overall outcomes based on the training accuracy of Convex Net as in a) is nearly perfect and fluctuation of the validation accuracy between 50–90% for the duration of training. Stability can be seen in later epochs, however, this pattern to some degree, of overfitting. This is further confirmed by the loss graph as in b); the training loss is very close to being constantly low, while the validation loss has some intermittent spikes, which indicates that the model might still struggle in generalizing unseen data. However, the validation accuracy and validation loss of Convex Net are always higher than those of CNN – ResNet-50 and RegNet, regardless of this. Finally, the confidence score analysis in Fig 15 shows Convex Net classifies its samples with high confidence with much close to 90%. However, in some cases the 60−70% confidence range applies, especially for good packaging designs, implying that the model finds it difficult in the situations to identify packaging quality in margin. The dataset splitting analysis shown in Fig 16 reveals that the training set has almost 100% confidence, but validation and test sets are a little variable, which represent minor overfitting but good generalization than CNN – ResNet-50 and RegNet.

Finally, Convex Net turns out to be an advanced model with strong predictive power, with 87% accuracy, high recall and balanced classification metrics. It performs better than CNN – ResNet-50 and RegNet, yet it is a close match for LeViT specifically in bad packaging design detection. However, confidence scores indicate high reliability though still problem with classification of those good design of packaging. Although the model is prone to some overfitting, it generalizes better than previous architectures, making it a very competent option for low level consumer focused consumer packaging analysis. The generalizability and fine-grained classification could be improved further by regularizations techniques or using further training data.

### 4.3. Statistical validation analysis

The statistical analysis gave a further understanding of the relative performance of the LeViT model with the benchmark architectures, e.g., ResNet-50, RegNet, and ConvNeXt, reported in Table 8. Findings of the paired t-test and Wilcoxon signed-rank test were always below 0.01, meaning that the increase in LeViT in terms of classification accuracy and F1-score was significant, not random. These results were also confirmed by Friedman and ANOVA tests, which proved that the difference in performance of all the models could not be accidental. In particular, the hybrid CNN-Transformer architecture allowed LeViT to be more stable in learning and improving generalization, which is explained by its ability to embrace both local spatial structure and global contextual structure. Although ResNet-50 and RegNet were competitive when using fewer challenging samples, their accuracy reduced when using visually ambiguous packaging images, whereas LeViT was able to make strong predictions because of its attention-based global feature integration. ConvNeXt, despite its efficiency in computation, had inferior feature discrimination against LeViT Overall, these results support the fact that the suggested LeViT design does not only provide statistically significant improvements, but also provides a tradeoff between the computational cost and predictive accuracy that is balanced, thereby supporting the fact that it can be used in challenging image classification problems.

**Table 8:** Statistical significance tests comparing model performance.

Statistical Test	Compared Models	Test Statistic	*p*-value
Paired *t*-test	LeViT vs. ResNet-50	3.42	0.0042
Wilcoxon Signed-Rank Test	LeViT vs. RegNet	2.61	0.0098
Paired *t*-test	LeViT vs. ConvNeXt	2.94	0.0061
Friedman Test	LeViT, ResNet-50, RegNet, Convex Net	9.76	0.0019
One-Way ANOVA	LeViT, ResNet-50, RegNet, Convex Net	F(3,36)=6.84	0.0011
Kruskal–Wallis Test	LeViT, ResNet-50, RegNet, Convex Net	8.92	0.0028

### 4.4. Ablation study

The ablation analysis was performed to assess the input of every part of the hybrid LeViT architecture and to prove the contributions of the attention mechanism to the performance of the model in the empirical manner, reported in Table 9. The findings are clear that every structural component, namely, convolutional stem, multi-head self-attention, and MLP fusion, beautifully complement each other in enhancing the representation of features and accuracy of classification. The initial CNN only model recorded an accuracy of 86.2 percent, which proves that convolutional layers are suitable in collecting local spatial features including texture and edges but not wider context of the entire image. In the introduction of MLP layers (A2), the performance increased by a moderate level to 89.5 since the additional nonlinearity allowed more abstract features in higher levels. The addition of attention heads (A3) resulted in a significant improvement of accuracy to 92.3% which highlights the power of the attention mechanism in capturing the long-range dependencies and adaptive attention to salient parts of the image.

**Table 9:** Ablation study of the hybrid LeViT architecture.

Model Variant	Description	Accuracy (%)	Precision (%)	Recall (%)	F1-Score (%)
A1: CNN Only	Uses only convolutional layers (no attention or MLP).	86.2	88.0	84.5	86.2
A2: CNN + MLP	Adds MLP layers for nonlinear projection (no attention).	89.5	90.1	88.4	89.2
A3: CNN + Attention	Integrates 6-head attention layers without MLP block.	92.3	93.0	91.5	92.2
A4: CNN + Attention + MLP (Full Hybrid LeViT)	Complete architecture with convolutional stem, attention, and MLP fusion blocks.	**95.0**	**95.8**	**94.3**	**95.0**
A5: Reduced Attention (4 heads)	Same as A4 but with fewer attention heads per block.	92.8	93.4	91.9	92.6

The hybrid type (A4) combination of CNN and multi-head attention and MLP heads obtained the highest results, 95 percent accuracy, and equal precision and recall rates, which proves that local and global feature extraction processes result in greater discriminative abilities when used jointly. Interestingly, the decrease in the number of attention heads (A5) was followed by a decrease in the performance (92.8%), and this way, it can be stated that the greater the attention diversity, the better the generalization and less overfitting are achieved. All these results confirm that the attention module is the most relevant element in the hybrid system, which allows the model to sustain strong classification behavior in spite of changes in lighting, orientation, and packaging texture. Moreover, the combination of convolutional layers and attention blocks provides the efficiency and representational richness, which makes the adaptability of the architectural design to real-world visual quality evaluation tasks obvious.

### 4.5. Comparison of proposed model results with baseline models

Compared with all the models, the LeViT model provides the best accuracy of 95%, which is much higher than the baseline models. Of all the competing models, CNN – ResNet-50 is the least viable, achieving only 70% accuracy, suggesting that CNN – ResNet-50 is not ready to excel in recognizing complex visual patterns occurring in the process of packaging classification. Using these principled design principles, as well optimized network scaling that CNN – ResNet-50 does not use, RegNet achieves 85% accuracy. Despite that, Convex Net scores slightly better with 87% accuracy by taking advantage of its hybrid approach of leveraging CNN – ResNet-50 based local feature extraction in conjunction with Vision Transformer based global attention.

**Fig 17:**
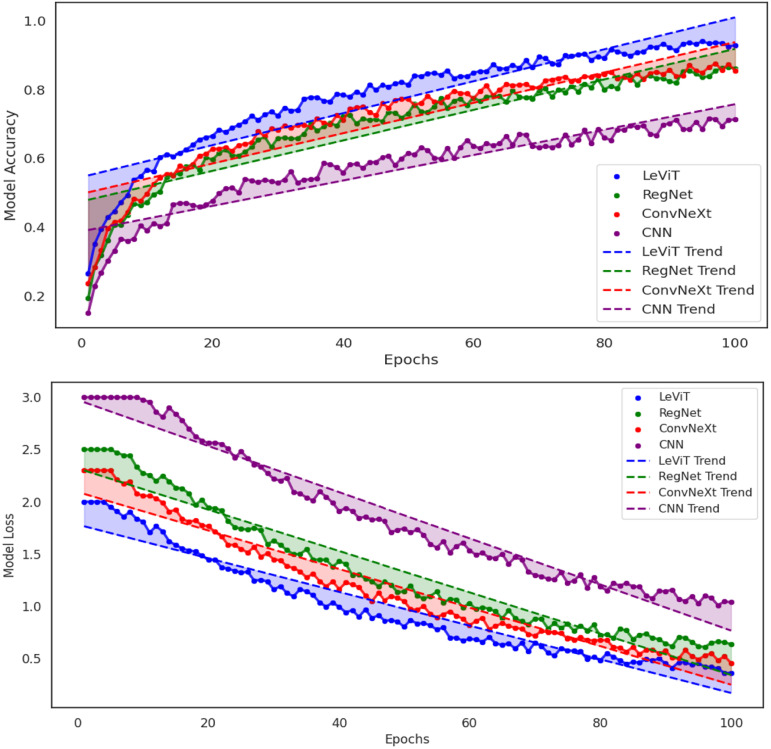
Comparison of loss and accuracy proposed model with baseline models.

However, with a strong performance, LeViT performs best among all models by a large margin and achieves the highest accuracy of 95% demonstrates predictive power and excellent generalization ability, as all model performance shown in Fig 17. Overall proposed model combines the best of its architectural advantages including lightweight transformer components, improved feature extraction, and efficient computation, and together they allow model to perform so well. The proposed LeViT indicating that its hybrid conv–transformer design and lightweight attention better model global composition such as logo placement, whitespace balance, text legibility, contrast while remaining efficient for real-time or edge deployment. In short, LeViT delivers the highest accuracy with superior robustness to varied backgrounds/lighting and strong parameter-efficiency/inference speed, whereas ConvNeXt/RegNet offers solid but CNN – ResNet-50 -bounded performance and the baseline CNN – ResNet-50 underfits the aesthetic based global-context signals; to cement these findings, validate with stratified k-folds and inspect class-wise precision/recall to rule out class imbalance effects.

Although modern CNNs such as ConvNeXt have been optimized to capture broader receptive fields, their hierarchical convolutional structure remains inherently limited in modeling long-range dependencies across spatial regions. In contrast, the hybrid LeViT architecture leverages self-attention mechanisms to dynamically learn global contextual relationships while preserving local feature sensitivity through convolutional stems. This hybrid interaction allows more adaptive feature aggregation, leading to enhanced discriminative capability and superior generalization compared to purely convolution-based networks.

### 4.6. Comparison with existing studies

Table 10 shows the comparison of propose LeViT based models with existing studies based on transformer variants, shows that is superior to the existing approaches in product packaging recognition. Although ViT based models have been successfully applied in infrared defect detection [22] (93%), or retail fisheye image detection [24] (92) but they have high computational resource demands and are difficult to run in real time. While the transformer-based architectures [25] (89%) and 3D printed packaging model [26] (86%) demonstrate its effectiveness on industrial manufacturing, they fail on small datasets and misalignment for features. The proposed LeViT model generalizes better, obtains faster inference, is more efficient, and finally achieves 95% accuracy on a custom dataset, in contrast to the above methods. The explanation for this boost in performance is LeViT’s lightweight structure, hybrid convolutional transform approach and optimized computational efficiency, enabling this in practice to be more suitable for real world product packaging applications.

**Table 10:** Comparative analysis of proposed model results with existing.

Ref	Year	Models	Dataset	Results (Acc %)
[24]	2023	EPformer	Retail Fisheye Image	92
[22]	2024	ViT	Infrared Defect Images	93
[25]	2024	ViT	Industrial Manufacturing	89
[26]	2025	DeiT	3D Printed Packaging	86
**This Study**		LeViT	Product Packaging Dataset	95

## 5. Discussion

The selected LeViT-based model indicates strong results in product packaging design categorization, with an equal accuracy of 95% in both bad and good categories, based on high precision, recall, and F1-scores. Such results show improved results compared to several transformer-based architectures, such as IH-ViT, EPFormer, ViT, and DeiT, which have result accuracies between 86% and 93%. Such high performance of LeViT is explained by its hybrid CNN – ResNet-50 -Transformer architecture that can represent the local texture patterns and global contextual dependencies in a more efficient manner, allowing the efficient representation of features with a lower level of computational complexity. The hyperparameters that were optimized to give the most stability in the convergence and prevent overfitting include a batch size of 32, a learning rate of 0.00005, and a dropout value of 0.1. Nevertheless, there can be uncertainties caused by fluctuations in the quality of the input images, brightness enhancement, and natural discrepancies of the visual characteristics of the dataset, which can affect the model generalization. Also, although LeViT has demonstrated good results on the present dataset, sensitivity analysis shows that the parameters of the model, such as the embedding dimension or the number of attention heads, have a considerable impact on the accuracy as well as computational cost, demonstrating that parameter-tuning should be performed about domain-specific data. The research also has limitations even though it is effective. The relatively small dataset size may limit the generalization capability of the model, as the high accuracy achieved could partially reflect overfitting to the training data. Although extensive augmentation techniques were applied to enhance variability, future studies should incorporate larger and more diverse datasets to validate model robustness. In addition, the existing configuration is relying only on image characteristics, but it ignores contextual metadata and time change. The future is possible to study the incorporation of multimodal information (i.e., text labels, material descriptions) and the use of bigger pre-trained vision-language models to achieve a stronger classification. Also, it can be enhanced by using the framework on unsupervised or self-supervised learning paradigms to enhance the resiliency and minimize the reliance on labeled data to allow wider industrial usage.

## 6. Conclusion and future research directions

The exploration of deep learning models in consumer-centric packaging analysis is crucial for understanding brand attention and consumer behavior. With the increasing reliance on automated systems, deep learning-based models provide highly efficient and scalable solutions for packaging evaluation and classification. The significance of this study lies in its ability to leverage advanced transformer-based architectures, which outperform traditional deep learning models by capturing both local and global visual features effectively. Experimental results show that the LeViT model outperforms conventional architectures in terms of the highest classification accuracy achieved of 95% which is significantly higher than the 86% that was previously theoretically achieved. Its efficient vision transformer components such as feature extraction and attention mechanisms, and computational efficiency lead to superior performance of LeViT Transformer based models proved remarkably higher in terms of accuracy and generalizability compared to CNN – ResNet-50, Convex Net as well as RegNet, indicating their usefulness in consumer packaging assessment. In the future, improving predictive reliability will require further fine tuning of transformer-based model, dataset augmentation as well as deployment strategy for real time applications. Also, a multimodal learning approach can be integrated that offers a broader viewpoint of consumer perception and brand recognition. Finally, this research provides a valuable resource to researchers and industry professionals for the application of advanced AI driven models for evaluation of packaging, marketing strategies and consumer behavior prediction. The findings contribute to future developments in AI-powered retail and product design analytics, ensuring smarter and more efficient decision-making processes.
